# Supplementary material for: A Regulatory Axis between Epithelial Splicing Regulatory Proteins and Estrogen Receptor α Modulates the Alternative Transcriptome of Luminal Breast Cancer
Source: Int J Mol Sci. 2022 Jul 16;23(14):7835. doi: 10.3390/ijms23147835 (PMC9319905; doi:10.3390/ijms23147835)
Supplement: Supplementary file 1 [file ijms-23-07835-s001.zip › Supplementary_Materials.pdf]

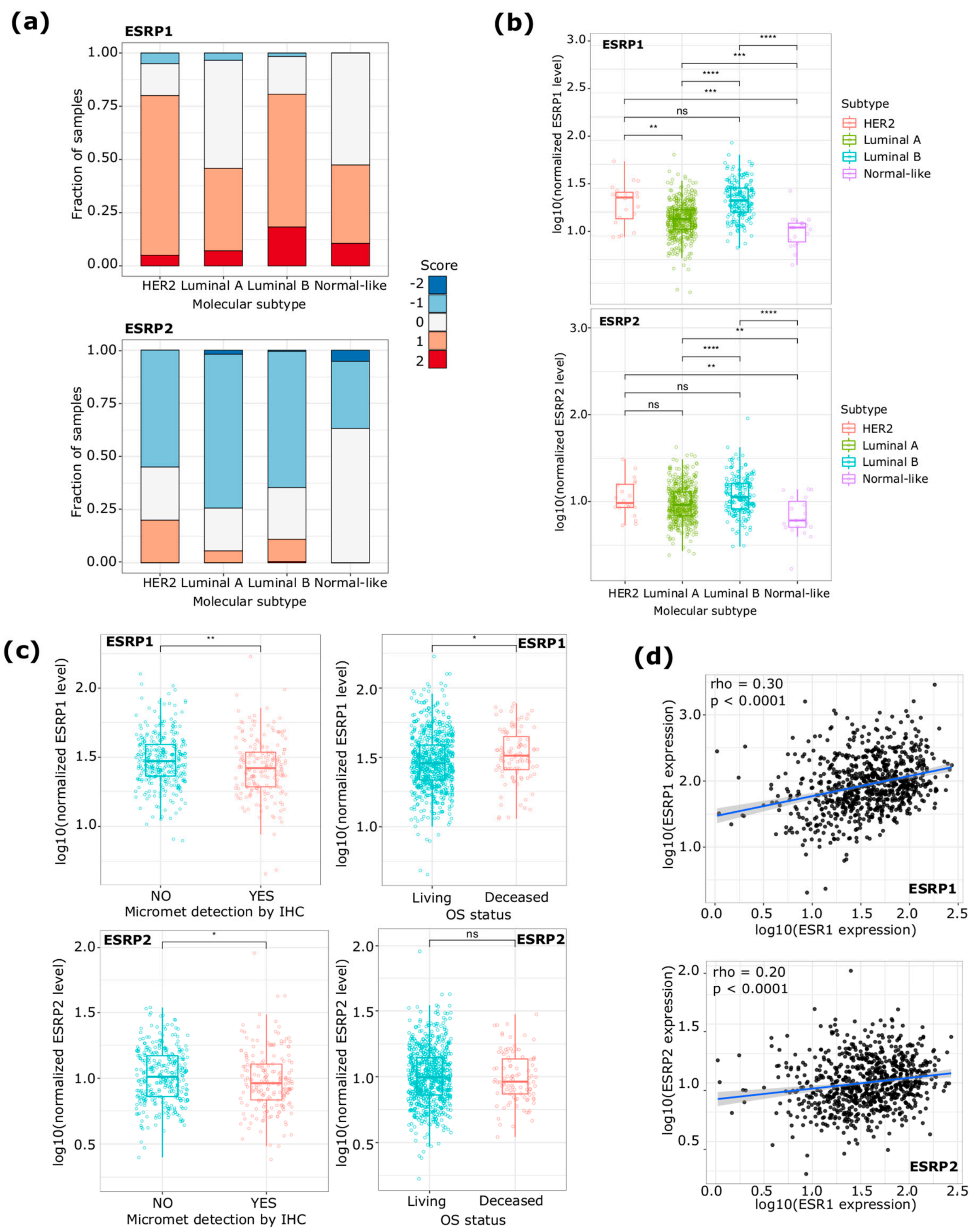

Supplementary Figure S1: **(a)** Box plots reporting the association between the fraction of samples analyzed and the copy number variation of *ESRP1* (left) and *ESRP2* (right) in distinct breast cancer molecular subtypes. (**b**) Box plots reporting the expression (y-axis) of *ESRP1* (top) and *ESRP2* (bottom) with respect to distinct intrinsic breast cancer subtypes (x-axis). ns, nonsignificant; **, $p < 0.01$; ***, $p < 0.001$; ****, $p < 0.0001$. **(c)** Box plots reporting the association between the levels of *ESRP1* (top) and *ESRP2* (bottom) and micrometastasis. ns, nonsignificant; *, $p < 0.05$; **, $p < 0.01$. (left) and overall survival for living and deceased patients, respectively. **(d)** Scatterplots showing the significant correlation between *ESRP1*, *ESRP2*, and *ESR1* mRNA levels in ERα+ breast cancers.

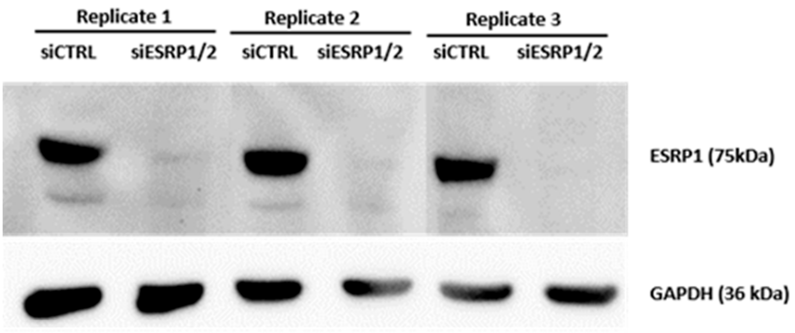

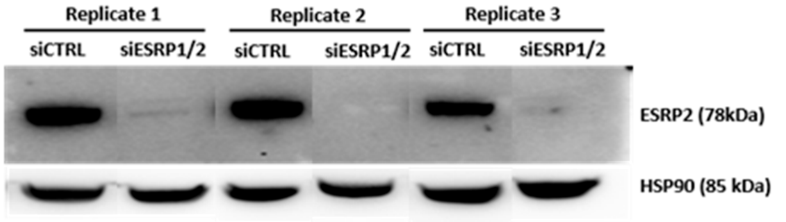

Supplementary Figure S2: Western blot validation of the efficacy of the transfection, indicating a significant downregulation of ESRP1 and ESRP2 proteins in MCF-7 BC cells transfected with ESRP1/2 combined siRNAs.

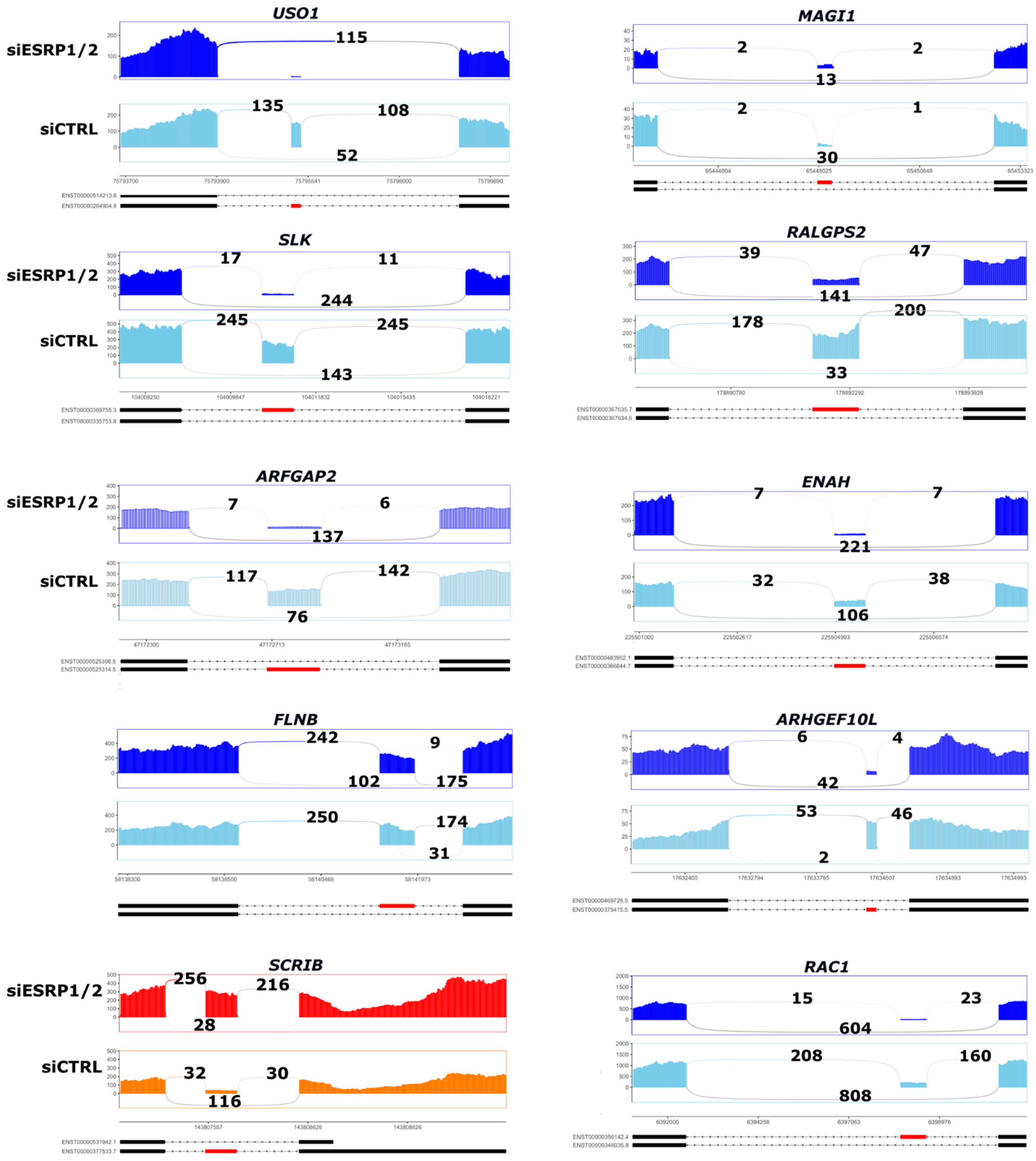

Supplementary Figure S3: changes in the EMT-related splicing signature upon ESRP1/2 silencing in MCF-7 cells. Sashimi Plots showing the expression changes of regulated exons and the junctions supporting their inclusion or exclusion events. The number of reads supporting each corresponding exon-exon junction is reported.

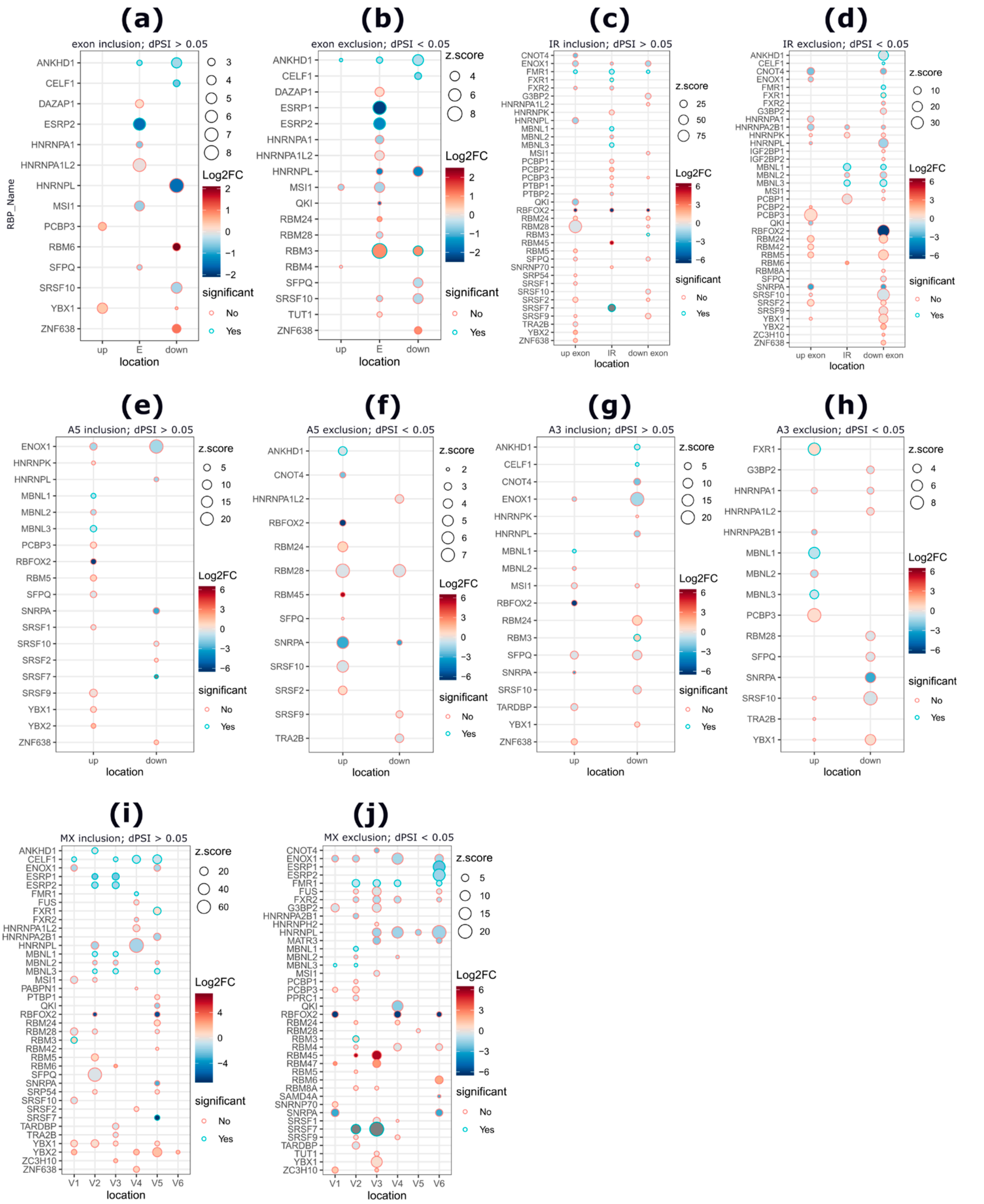

Supplementary Figure S4: RBP binding motif enrichment analysis performed on sequences involving **(a,b)** exon skipping, intron retention **(c,d)**, splice site selection A5′ **(e,f)**, A3′ **(g,h)**, and MXE **(I,j)** events upon ESRP1/2 silencing. Scanned sequences are regulated exons extended 200nt on both sides, except in MXE events where the extension is of 100nt on both sides of the mutually exclusive exons (see Materials and Method section). The size of the dotes is proportional to the enrichment z-score, which represents the occurrence of an RBP's binding motif in a regulated ASE as compared to that in control events. up and down location refers to 200nt of the flanking exons upstream and downstream the retained intron, respectively. **ES**, Exon Skipping; IR, Intron Retention; **A3′SS**, Alternative 3′ Splice Sites; **A5′SS**, Alternative 5′ Splice Sites; **MXE**, Mutually Exclusive Exons.

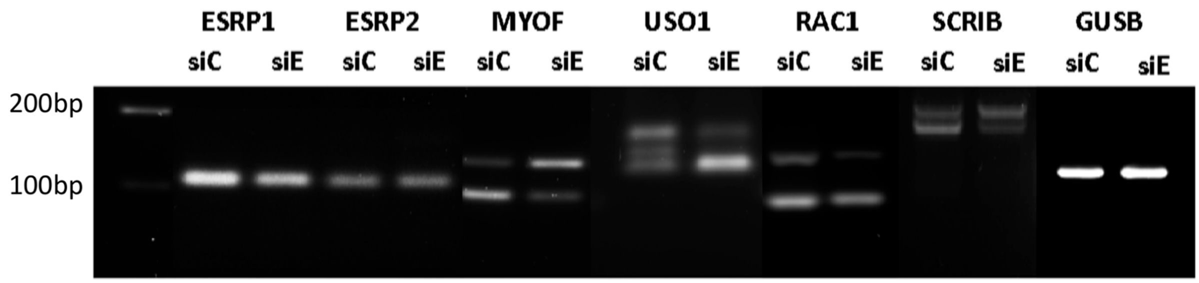

Supplementary Figure S5: Complete agarose gel electrophoresis from the RT-PCR validation of ASE. siC = control siRNA, siE = ESRP1/2-targeting siRNA.

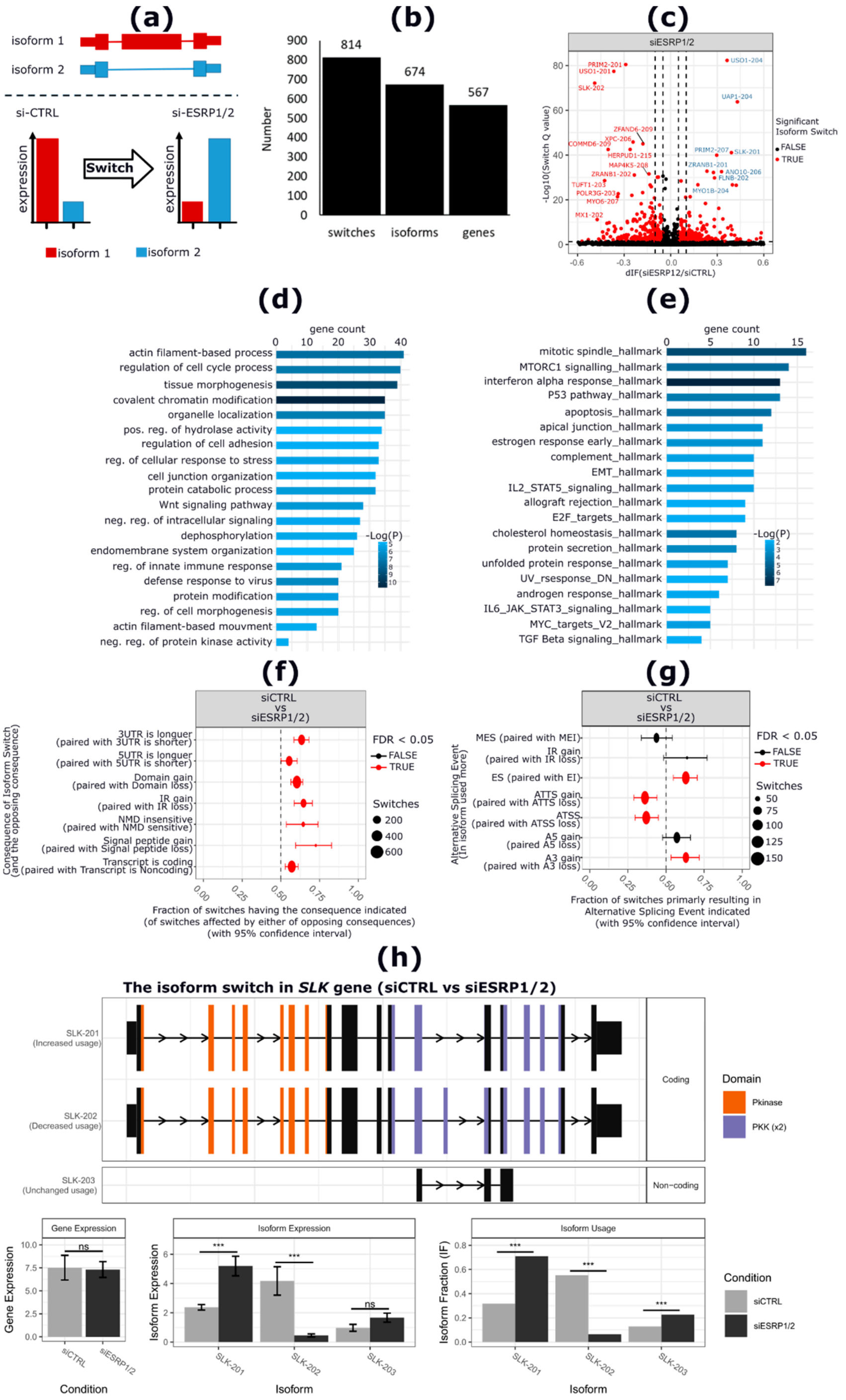

Supplementary Figure S6: (a) Isoform switching analysis of ESRP1/2 silencing experiment. **(b)** Numbers of significant switching events and isoforms/genes involved. **(c)** Volcano plot showing the differential isoform fraction (dIF) and BH-adjusted *p*-values of switching events. **(d-e)** Enriched biological processes and gene set hallmarks of genes with significant switching isoforms. **(f)** Functional consequences of switching events (and the proportion of switches (x-axis) having either of the opposing consequences. **(g)** AS events (ASEs)predicted to be enriched and the proportions (x-axis) of switches resulting from either opposing ASEs. **MES**, Multiple Exon Skipping; **IR**, Intron Retention; **ES**, Exon Skipping; **ATTS**, Alternative Transcription Termination Site; **ATSS**, Alternative Transcription Start Site; **A5**, Alternative A5′ Splice Site; **A3**, Alternative 3′ Splice Site. (h) SLK switching isoform pairs resulting from ESRP1/2 combined silencing. ns, not significant; ***, $p < 0.0001$.

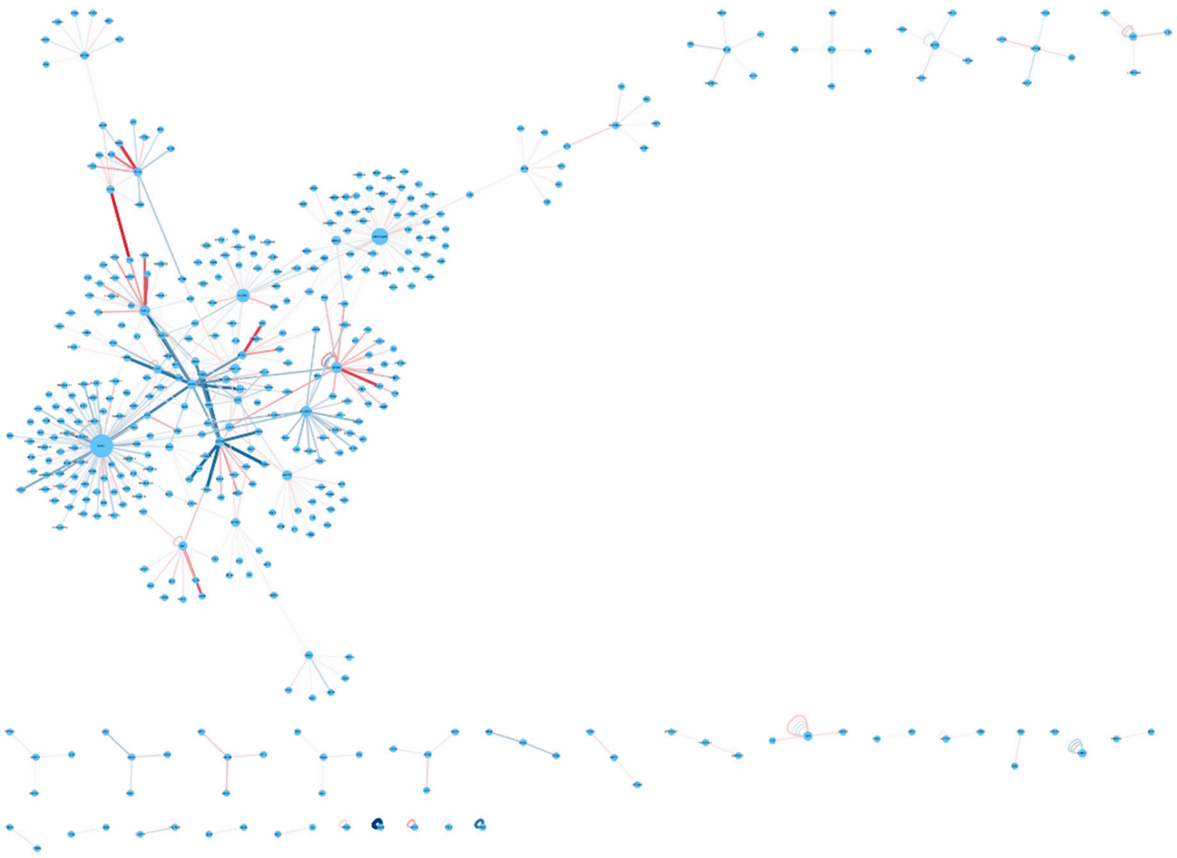

Supplementary Figure S7: Complete PPI subnetwork constructed starting from the 95 genes that harbored significant ES events, and whose inclusion/exclusion levels correlated ($p < 0.05$) with ESRP1 and ESRP2 mRNA levels in ERα+ BCs. Size of each node is proportional to its degree (range = 1, 80). Positive and negative dIS values are represented by the edge's color in red and blue, respectively, and the width of edges is proportional to the absolute value of dIS.

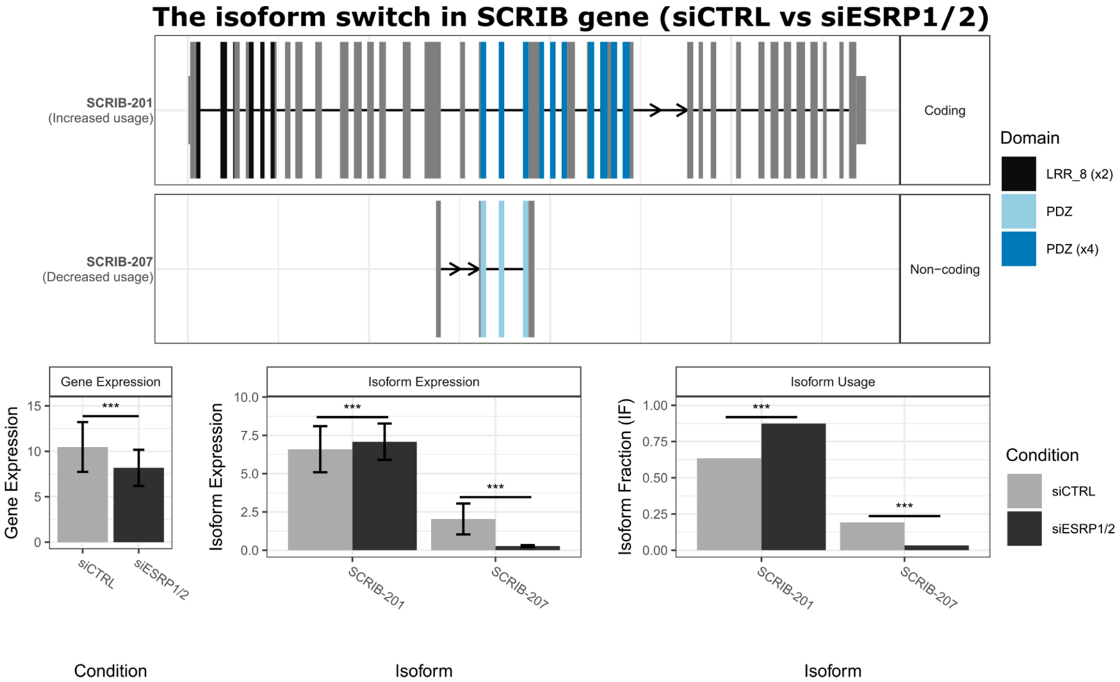

Supplementary Figure S8: SCRIB switching isoform pairs resulting from ESRP1/2 combined silencing. The two isoforms and their exon and protein domains are illustrated. Blue and Black colors of the exons indicate the LRR_8 and PDZ protein domains, respectively. Boxplots indicate the differential regulation of SCRIB at gene and isoform levels. ***, $p < 0.0001$.

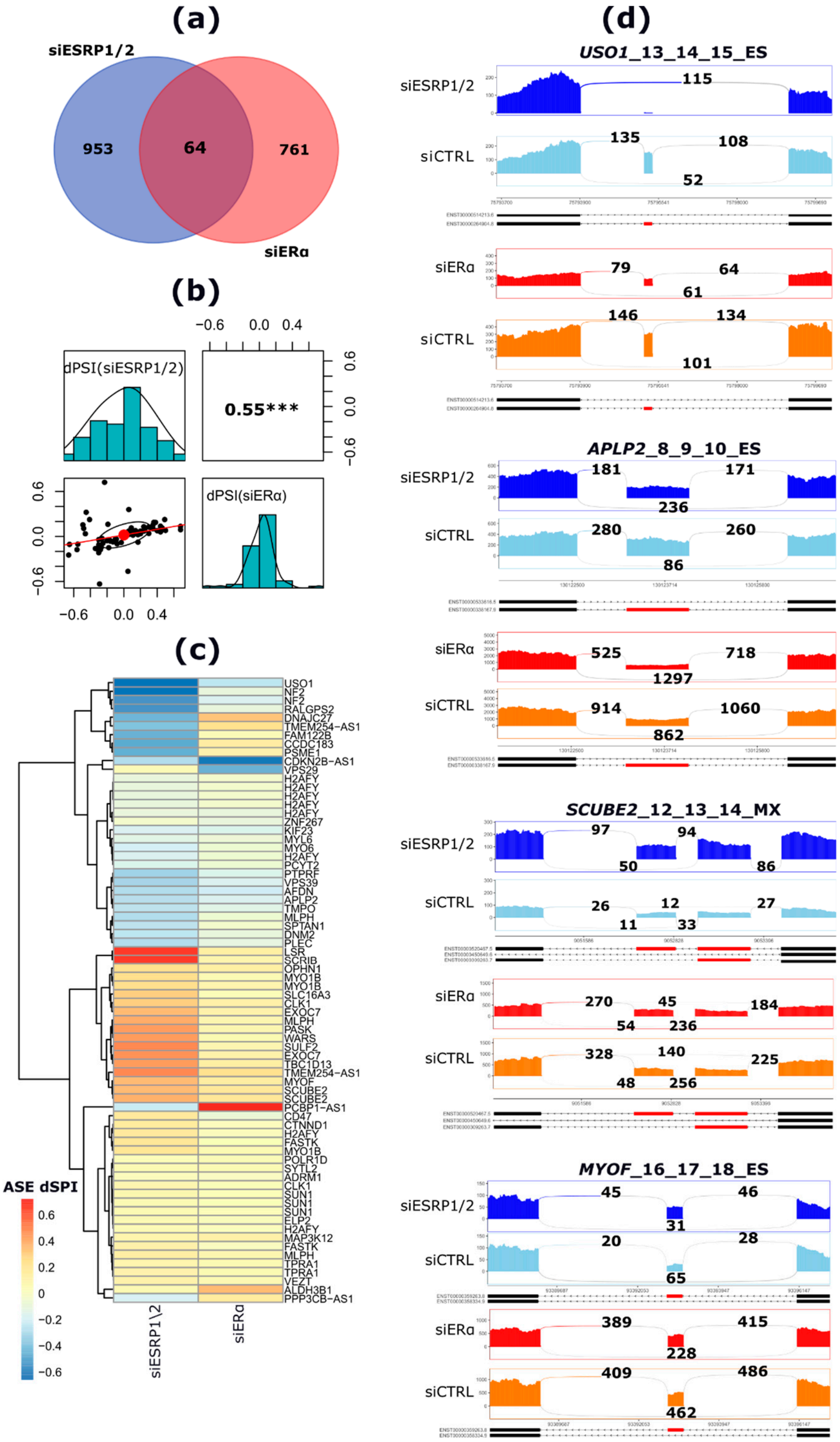

Supplementary Figure S9: An overview of ASEs regulated upon both siER*α* [1] and si-ESRP1/2 in MCF-7 BC cells. **(a)** Venn diagram reporting the number of ASEs overlapping both datasets. ***, $p < 0.001$. **(b)** Correlation plot showing the correlation between the dPSI values of the 64 overlapping events shown in (a). **(c)** heat map showing the direction of splicing changes (dPSI values) of the overlapping ASEs. **(d)** sashimi plots of selected examples of ASEs regulated in both datasets. Numbers representing the normalized read counts supporting either inclusion or exclusion of regulated exons (highlighted in red colors) are reported. Isoforms concerned by the ASEs are also shown.

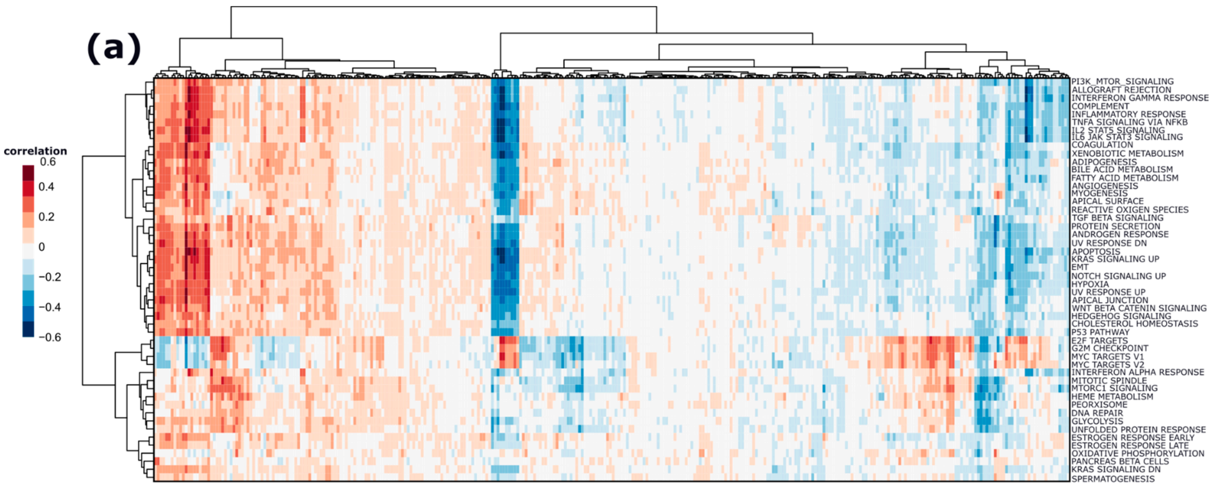

Supplementary Figure S10: (a) Heat map plot showing the correlation between the expression of ASEs regulated by ESRP1/2 silencing in MCF-7 cells and related molecular pathways in ERα+ tumors.

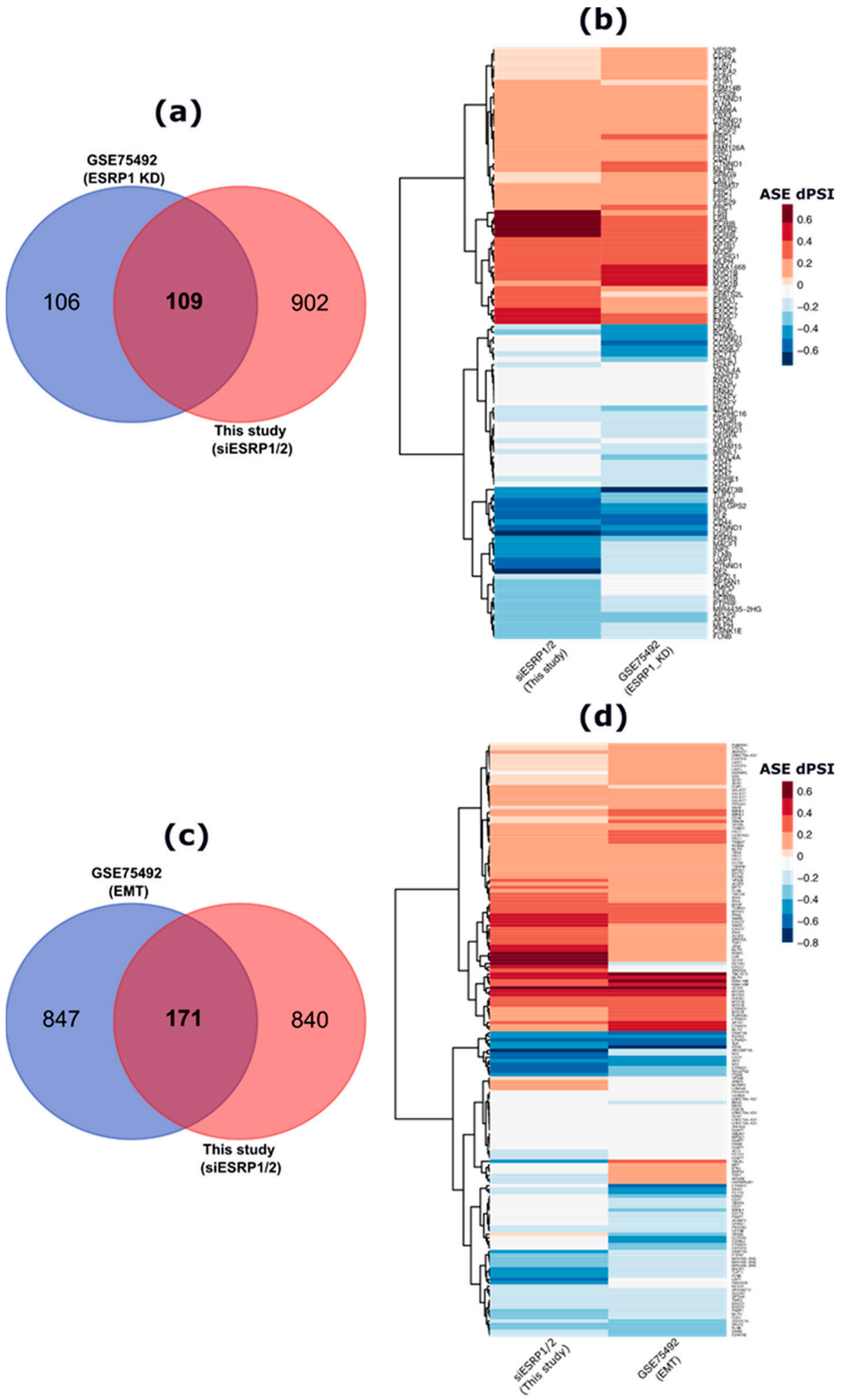

Supplementary Figure S11: Overview of the overlap in AS changes between this study and the (GSE75492) study. **(a)** Venn diagram showing the number of overlapping ASEs between this study (siESRP1/2) and the ESRP1 knock down (ESRP1 KD) experiment by [2]. **(b)** Heat map plot reporting the dPSI of the 109 overlapping ASEs shown in (a). **(c)** Venn diagram representing the number of overlapping ASEs between this study and the EMT induction experiment by [2]. **(d)** Heat map plot representing the dPSI of the 171 overlapping ASEs shown in (c).

References

1. Elhasnaoui, J.; Ferrero, G.; Miano, V.; Cutrupi, S.; De Bortoli, M. The Estrogen Receptor α Signaling Pathway Controls Alternative Splicing in the Absence of Ligands in Breast Cancer Cells. *Cancers* **2021**, *13*, doi:10.3390/cancers13246261.
2. Yang, Y.; Park, J.W.; Bebee, T.W.; Warzecha, C.C.; Guo, Y.; Shang, X.; Xing, Y.; Carstens, R.P. Determination of a Comprehensive Alternative Splicing Regulatory Network and Combinatorial Regulation by Key Factors during the Epithelial-to-Mesenchymal Transition. *Molecular and Cellular Biology* 2016, *36*, 1704–1719.
